# Construction of a New *Agrobacterium tumefaciens*-Mediated Transformation System based on a Dual Auxotrophic Approach in *Cordyceps militaris*

**DOI:** 10.4014/jmb.2312.12003

**Published:** 2024-03-18

**Authors:** Huan huan Yan, Yi tong Shang, Li hong Wang, Xue qin Tian, Van-Tuan Tran, Li hua Yao, Bin Zeng, Zhi hong Hu

**Affiliations:** 1 College of Life Sciences, Jiangxi Science and Technology Normal University, Nanchang 330013, P.R. China; 2 Jiangxi Key Laboratory of Bioprocess Engineering, Jiangxi Science and Technology Normal University, Nanchang 330013, P.R. China; 3 Shenzhen Technology University, Shenzhen 518118, P.R. China; 4 VNU University of Science, 334 Nguyen Trai, Thanh Xuan, Hanoi, Vietnam

**Keywords:** *Cordyceps militaris*, *Agrobacterium tumefaciens*-mediated transformation, uridine/uracil auxotrophic mutant, histidine auxotrophic mutant, genetic modification

## Abstract

*Cordyceps militaris* is a significant edible fungus that produces a variety of bioactive compounds. We have previously established a uridine/uracil auxotrophic mutant and a corresponding *Agrobacterium tumefaciens*-mediated transformation (ATMT) system for genetic characterization in *C. militaris* using *pyrG* as a screening marker. In this study, we constructed an ATMT system based on a dual *pyrG* and *hisB* auxotrophic mutant of *C. militaris*. Using the uridine/uracil auxotrophic mutant as the background and *pyrG* as a selection marker, the *hisB* gene encoding imidazole glycerophosphate dehydratase, required for histidine biosynthesis, was knocked out by homologous recombination to construct a histidine auxotrophic *C. militaris* mutant. Then, *pyrG* in the histidine auxotrophic mutant was deleted to construct a Δ*pyrG* Δ*hisB* dual auxotrophic mutant. Further, we established an ATMT transformation system based on the dual auxotrophic *C. militaris* by using *GFP* and *DsRed* as reporter genes. Finally, to demonstrate the application of this dual transformation system for studies of gene function, knock out and complementation of the photoreceptor gene Cm*WC-1* in the dual auxotrophic *C. militaris* were performed. The newly constructed ATMT system with histidine and uridine/uracil auxotrophic markers provides a promising tool for genetic modifications in the medicinal fungus *C. militaris*.

## Introduction

*Cordyceps militaris* is an entomopathogenic filamentous fungus belonging to the phylum *Ascomycota*. It is a traditional Asian medicinal material with high medicinal and nutritional value [1-3]. *C. militaris* contains a variety of secondary metabolites, including cordycepin (3'-deoxyadenosine) and its derivatives, carotenoids, and polysaccharides. These metabolites show significant anti-tumor, anti-cancer, antioxidant, anti-inflammatory, antibacterial, anti-aging, insecticidal, lipid-lowering, hypoglycemic, and immune regulatory effects [4-7]. *C. militaris* mainly parasitizes the pupae of lepidopteran insects. Compared with *C. sinensis*, *C. militaris* grows faster and can produce fruiting bodies by artificial culture. Therefore, *C. militaris* is often exploited as an ideal substitute for the widely studied *C. sinensis* [8, 9]. Recent studies have shown that cordycepin is one of the key active components in *C. militaris*, and this compound may contribute to the therapeutic and preventive effects of the species against various diseases [10]. The genome sequence of *C. militaris* has been reported [11], and increasing research has focused on molecular and cell biological processes in the species. Genetic transformation systems are important for molecular research. Common methods for fungal transformation include *Agrobacterium tumefaciens*-mediated transformation (ATMT), polyethylene glycol (PEG)-mediated protoplast transformation (PMPT), electroporation, and gene gun-mediated transformation. In ATMT, *A. tumefaciens* infects the host cell and T-DNA of the recombinant plasmid is integrated into the host genome [12]. In PTMT, chemical reagents facilitate the fusion of exogenous DNA with protoplasts, and the transformed protoplasts regenerate the cell wall, resulting in exogenous gene transformation [13]. Electroporation is based on the reversible permeation of the biomembrane induced by short-term, high-amplitude electric fields during electrical pulses, allowing for the absorption of recombinant DNA into the host cell [14]. Gene gun-mediated transformation uses an acceleration device to introduce particles coated with exogenous genes into recipient cells and tissues. Then, the foreign gene is integrated into the genome of recipient cells [15]. Compared with gene gun-mediated transformation and electroporation, ATMT and PTMT are simpler and more practicable [16, 17] and thus are more widely used. ATMT is relatively easy; it only requires the co-cultivation of spores and *A. tumefaciens* carrying the target gene, followed by selection in a screening medium [18]. The target gene is randomly inserted into the genome and inherited stably during recipient cell division. Moreover, the transformation efficiency is high.

Selectable markers are also important in genetic manipulation. If a strain is sensitive to antibiotics, corresponding resistance genes as selection markers can effectively reduce the workload involved in screening transformants [19]. Auxotrophic mutants are commonly used for genetic transformation. Ura3/*pyrG* encoding orotidine-5'-phosphate decarboxylase, required for uridine (the precursor of uracil) biosynthesis, is commonly used as an auxotrophic screening marker in fungal genetic transformation. Cells can only grow on culture medium with exogenous uracil or uridine after the mutation or knockout of this gene [20]. In addition, orotidine-5'-phosphate decarboxylase encoded by the *pyrG* gene can catalyze 5-fluoroorotic acid (5-FOA) to synthesize 5-fluorouracil monophosphate, which shows toxicity [21], inhibiting the growth of wild-type strains in medium containing 5-FOA. ATMT systems using *pyrG* as a screening marker have been established in *Aspergillus oryzae* and other fungi, providing a new approach to genetic transformation [22-24]. The biosynthesis of histidine is essential for the growth and development of fungi and other microorganisms, and *imidazole glycerophosphate dehydratase* (*IGPD*), required for the biosynthesis of histidine, is commonly used for the construction of histidine auxotrophic fungal strains. The histidine auxotrophic marker has also been used as a selection marker for screening filamentous fungi transformants [25]. The *hisB* gene, which is essential for histidine biosynthesis, is a reliable selection marker for genetic transformation in *A. oryzae* [26].

In a previous study, we constructed a *pyrG* auxotrophic strain in *C. militaris* [27]. In this study, we generated histidine auxotrophic (*hisB*) and *pyrG* *hisB* double auxotrophic strains. Furthermore, we constructed an ATMT system based on the dual auxotrophic mutant with two screening markers, *hisB* and *pyrG*. By using these screening markers, we achieved the co-expression of *DsRed* and *GFP* fluorescent reporter genes in the dual auxotrophic mutant of *C. militaris*. We also knocked out the photoreceptor gene Cm*WC-1* and complemented the mutant using the dual auxotrophic *C. militaris* as the background to confirm the efficiency of the transformation system. Our ATMT system provides a promising genetic tool for studying the regulation of metabolism and gene functions in *C. militaris*.

## Microbial Strains and Plasmids

The strains, plasmids, and primers used in this study are listed in Table S1 and Table S2. The wild-type *C. militaris* strain was isolated by our team and was used for DNA extraction. CmΔ*pyrG* was used as the background strain for the construction of the histidine auxotrophic strain (CmΔ*hisB*) and CmΔ*pyrG* Δ*hisB* double mutant. *Escherichia coli* DH5α and *A. tumefaciens* AGL1 were used for target plasmid construction and fungal transformation, respectively.

### Medium for Fungal Cultivation and Genetic Transformation

LB medium with the corresponding antibiotic was used to culture *E. coli* for plasmid construction and *A. tumefaciens* for *C. militaris* transformation. The co-culture process for ATMT was carried out by using induction medium (IM), and the transformants were screened using Czapek-Dox (CD) medium. The specific formulations of the media are described previously [27].

### Identification of a Histidine Biosynthesis Gene

The amino acid sequence for the histidine biosynthesis gene *IGPD* in *C. militaris* was queried by searching against the NCBI (https://www.NCBI.nlm.nih.gov/) database to obtain reference sequences from *A. oryzae* (XP_001822962.1) and homologous sequences in *A. niger* (KAI2995965.1), *A. fumigatus* (KAH1277580.1), *A. nidulans* (AAK15457.1), and *S. cerevisiae* (KAF4004652.1). A phylogenetic analysis of selected amino acid sequences was carried out using MEGA-X (neighbor-joining). A multiple sequence alignment of *IGPD* proteins from different fungi was generated using DNAMAN.

### Construction of Histidine Auxotrophic *C. militaris*

The CmΔ*hisB* vector was used to knockout the *hisB* gene in *C. militaris* by *A. tumefaciens*-mediated transformation. The background strain CmΔ*pyrG* was constructed in our previous study [27]. For construction of the CmΔ*hisB* vector, wild-type *C. militaris* DNA was used as a template to amplify the 5' flanking region (1294 bp) and 3' flanking region (1244 bp) of *hisB* with the primers *hisB* upstream F1/R1 and *hisB* downstream F2/R2 (Table S2) and the binary vector pEX1 (with the *A. oryzae* *pyrG* expression cassette as a selection marker) used for *A. oryzae* transformation was selected as a skeleton. Then, the 3' flanking region was inserted into the left border of the *pyrG* expression cassette using *Spe*I and *Hin*dIII, and the 5' flanking region was inserted into the right border of the *pyrG* expression cassette using *Eco*RI. The ligation of target fragments into linearized plasmids was conducted by using a one-step cloning kit (Vazyme Biotech Co., Ltd., China). Then, CmΔ*hisB* was transformed into *A. tumefaciens*, followed by *C. militaris* transformation following previously described methods, with some modifications [27]. *A. tumefaciens* containing the CmΔ*hisB* vector was co-cultured with spores of CmΔ*pyrG* *C. militaris* on IM (containing 40 mmol/l MES and 200 μmol/l AS) for 60 h and then screened on CD with histidine supplementation (containing 300 μg/ml cefotaxime). Finally, the transformants were cultured on CD medium and CD + histidine medium to observe the phenotype and verified by PCR with the primers CmΔ*his* F/R (Table S2).

### Transformation System Using *hisB* as the Selection Marker

The pEX2D binary vector (with the *A. oryzae* *hisB* expression cassette as a selection marker and *DsRed* fluorescent reporter genes) was used to transform CmΔ*hisB* *C. militaris*. We have previously shown that the glyceraldehyde 3-phosphate dehydrogenase promoter P*gpdA* and α amylase promoter *PamyB* in *A. oryzae* could not drive the expression of *GFP* or *DsRed* reporters in *C. militaris* [27]. Thus, the promoter of the reporter gene in pEX2D was replaced with the IF promoter, the promoter of the extension factor, in *C. militaris* to construct the IF-pEX2D vector [26, 27]. Then, IF-pEX2D was transformed into *A. tumefaciens* and *C. militaris*. The transformants were identified by PCR verification using the primers CmΔ*his* F/R and *DsRed* F/R (Table S2) and fluorescence observation.

### Construction of Uridine Histidine Dual Auxotrophic *C. militaris*

During the construction of the Δ*hisB* mutant, the ORF region of *hisB* was replaced with the *pyrG* expression cassette. Thus, we took advantage of homologous recombination to knock out the *pyrG* expression cassette to construct the Δ*pyrG* Δ*hisB* double mutant. The 5' and 3' flanking regions of *hisB* used for *hisB* deletion were fused by PCR. The fragment was inserted into the pEX1 plasmid linearized by *Eco*RI and *Hin*dIII to remove unnecessary elements and construct CmΔ*pyrG*. Then, the plasmid was transformed into *C. militaris* by ATMT. To facilitate gene transfer, 0.02% histidine was added to the IM (MES containing 40 mmol/l and AS 200 μmol/l). To screen the CmΔ*pyrG* Δ*hisB* double mutant, CD screening medium was supplemented with 2% uridine, 2% uracil, 2% histidine, 300 μg/ml cefotaxime, and 1% 5-FOA. The transformants were transferred to CD + his, CD + Uri/Ura + his, and CD + Uri/Ura + his to observe phenotypes, followed by confirmation by PCR amplification.

### Transformation of *hisB* and *pyrG* as Selection Markers

Using the binary vector IF-pEX1 with the *pyrG* expression cassette and *GFP* reporter gene in a dual auxotrophic mutant of *C. militaris* (CmΔ*pyrG*Δ*hisB*), the co-culture medium was supplemented with 0.02% histidine, 0.02%uridine, 0.02% uracil, 40 mmol/l MES, and 200 μmol/l AS and screened using CD + his medium (containing 300 μg/ml cefotaxime). After the plates were cultured at 22°C for 7–10 days, transformants were transferred to CD+ his medium to observe their growth state. The *GFP* gene was molecularly verified, and the *GFP* signal was observed under a fluorescence microscope. The *DsRed* gene was expressed by the binary vector IF-pEX2D with the *hisB* marker. The medium was supplemented with 0.02% histidine, 40 mmol/L MES, and 200 μmol/l AS. The medium was screened with CD + cef (300 μg/ml cefotaxime). The transformants of *C. militaris* with *GFP* and *DsRed* reporter genes were obtained and cultured on CD, CD + Uri/Ura, CD + his, CD + Uri/Ura + his medium, and the phenotypes of CmΔ*pyrG*Δ*hisB*, *pyrG*-*GFP*, *hisB*-*DsRed*, and *pyrG*-*GFP*-*hisB*-*DsRed* were observed. Fluorescent gene expression in the transformants was examined by fluorescence microscopy.

### Deletion and Complementation of Cm*WC-1* in *C. militaris*

Like the knockout of the *hisB* gene, the 5' and 3' flanking sequences of Cm*WC-1* (AGO64764.1) were amplified and fused to the left and right side of the *pyrG* expression cassette in the pEX1 vector. The primers are listed in Table S2, and the constructed vector was named CmΔWC-1. Then, the plasmid CmΔWC-1 was transferred into the *C. militaris* dual-deficient strain CmΔ*pyrG* Δ*hisB* by ATMT. The knockout mutant was identified based on the growth phenotype and PCR amplification using the primers CmΔWC-1 F/R (Table S2). The complementary vector IF-Cm*WC-1* was constructed using the binary vector IF-pEX2D carrying the *hisB* marker. Similarly, the transformation of *C. militaris* was carried out by ATMT. The transformants were confirmed by the growth phenotype and PCR amplification.

## Results

### *C. militaris* Possesses a Conserved *hisB* Gene

**Fig. 1:**
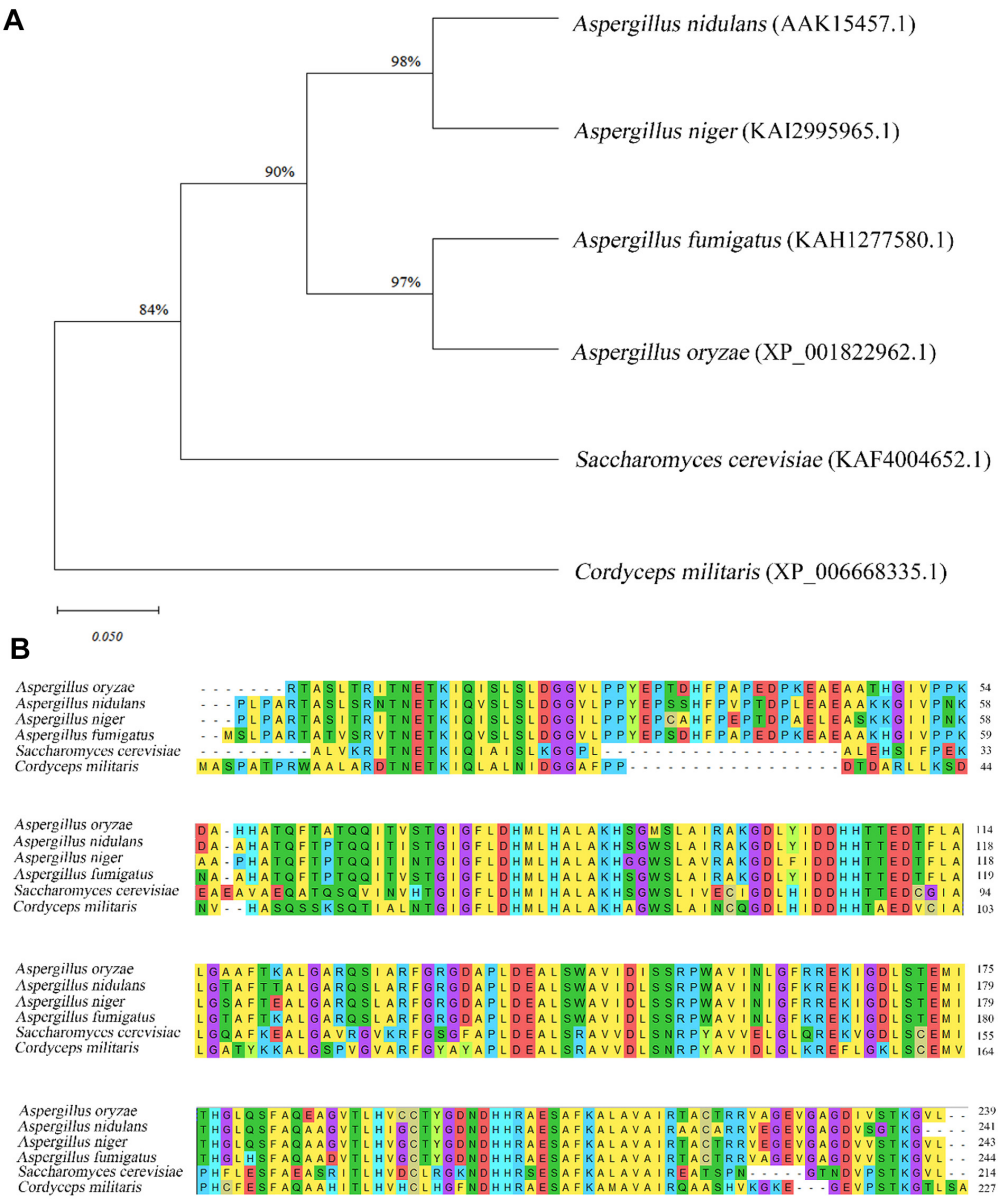
Phylogenetic tree of *hisB* in different fungi and comparison of protein sequences. (**A**) MEGA-X was used to construct the phylogenetic tree based on imidazole glycerophosphate dehydratase proteins of different fungi. (**B**) Amino acid sequence alignment of imidazole glycerophosphate dehydratase in different fungi.

IGPD, required for histidine biosynthesis, was identified in the *C. militaris* genome. Using the *S. cerevisiae* histidine biosynthesis gene *IGPD* (KAF4004652.1) as a reference sequence, a single copy of the homologous gene (XP_006668335.1) was found in the *C. militaris* genome using BLAST. *IGPD* genes (hereafter *hisB*) in other fungi, including *A. oryzae*, *A. niger*, *A. fumigatus*, and *A. nidulans*, were also obtained using BLAST searches. A phylogenetic analysis showed that the *hisB* gene in *C. militaris* was more similar to that in *S. cerevisiae* than to homologues in *Aspergillus* species (Fig. 1A). The amino acid sequence was also conserved among these species, with an amino acid sequence identity of 87.41%. Therefore, the *C. militaris* genome had a highly conserved *hisB* gene.

### Deletion of *hisB* in *C. militaris* Using *pyrG* as a Screening Marker

**Fig. 2:**
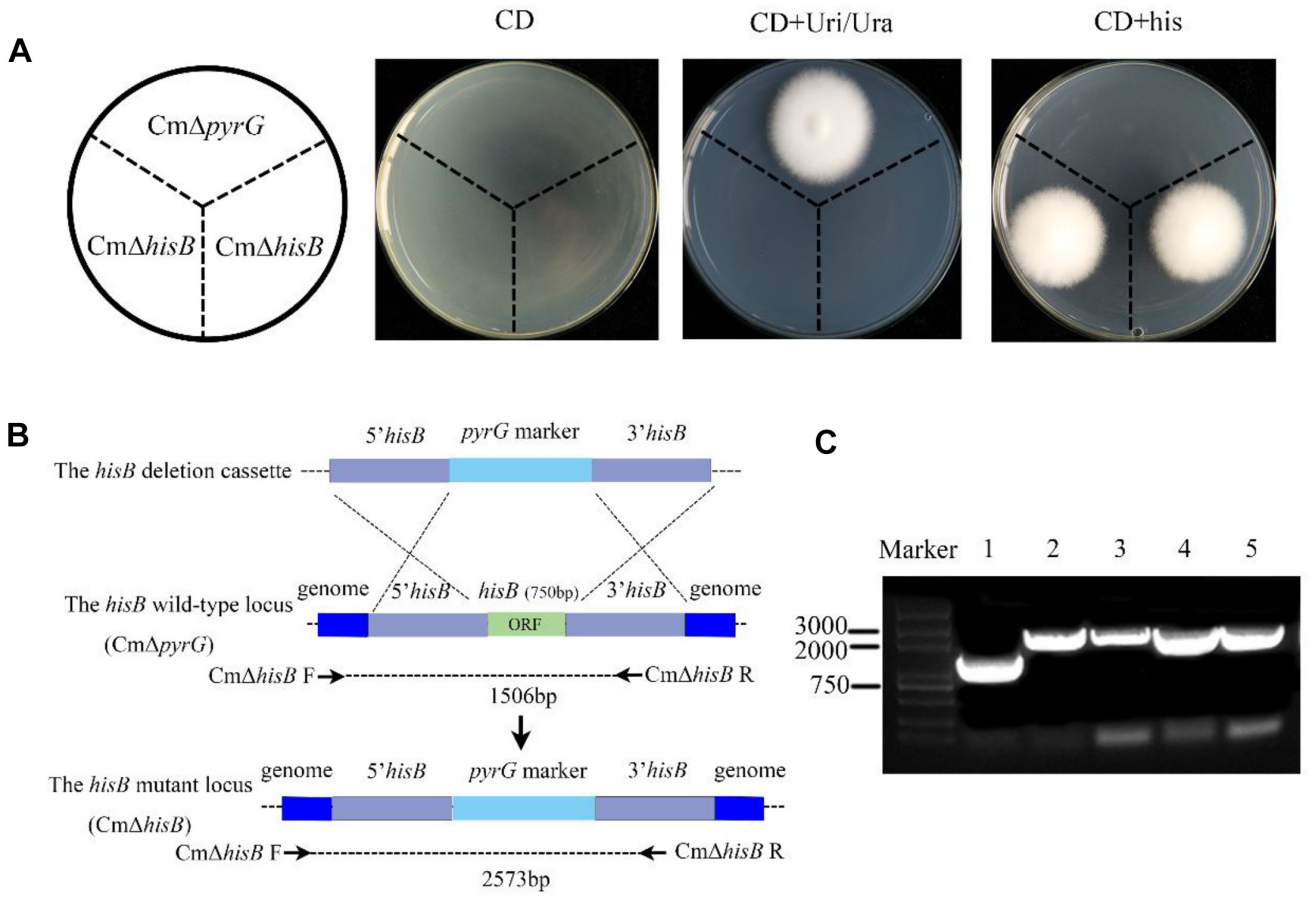
Construction of a histidine auxotrophic mutant of *C. militaris*. (**A**) Phenotypes of the background strain CmΔ*pyrG* and *hisB* auxotrophic mutant (CmΔ*hisB*) cultured on CD, CD + Uri + Ura, and CD + his medium. Spores of each type of *C. militaris* were added to the medium and cultured at 22°C for 6–8 days to confirm the deletion of *hisB* in *C. militaris*. (**B**) Knockout of *hisB* in *C. militaris*. (**C**) PCR analysis using primers CmΔ*hisB* F/R confirmed the deletion of *hisB* in positive strains: line 1 is the wild-type control; lines 2–5 are histidine-deficient transformants.

We used the CmΔ*pyrG* strain maintained in our lab [27] as the background to construct histidine auxotrophic *C. militaris* by homologous recombination. The 5' and 3' flanking regions of the ORF of *hisB* were fused to the left and right border of the *pyrG* expression cassette in pEX1 to construct the CmΔ*hisB* vector (Fig. 2B). The constructed CmΔ*hisB* vector was transformed into CmΔ*pyrG* by ATMT. Transformants that only grew in CD medium with exogenous histidine were selected for further analyses. As shown in Figure 1, the background strain, CmΔ*pyrG*, only grew on the CD medium with exogenous Uri/Ura. The CmΔ*hisB* mutant only grew in CD medium with exogenous histidine (Fig. 2A). In the case of non-homologous recombination leading to an ectopic insertion, a fragment of 1506 bp was amplified. With homologous recombination, the band size was 2573 bp (Fig. 2B). Furthermore, using PCR sequencing, we validated that the target gene was successfully knocked out (Fig. 2C). Thus, we constructed a histidine auxotrophic *C. militaris* using the *pyrG* expression cassette to replace *hisB* in the CmΔ*pyrG* background.

### Construction of a Transformation System Using *hisB* as a Selection Marker

**Fig. 3:**
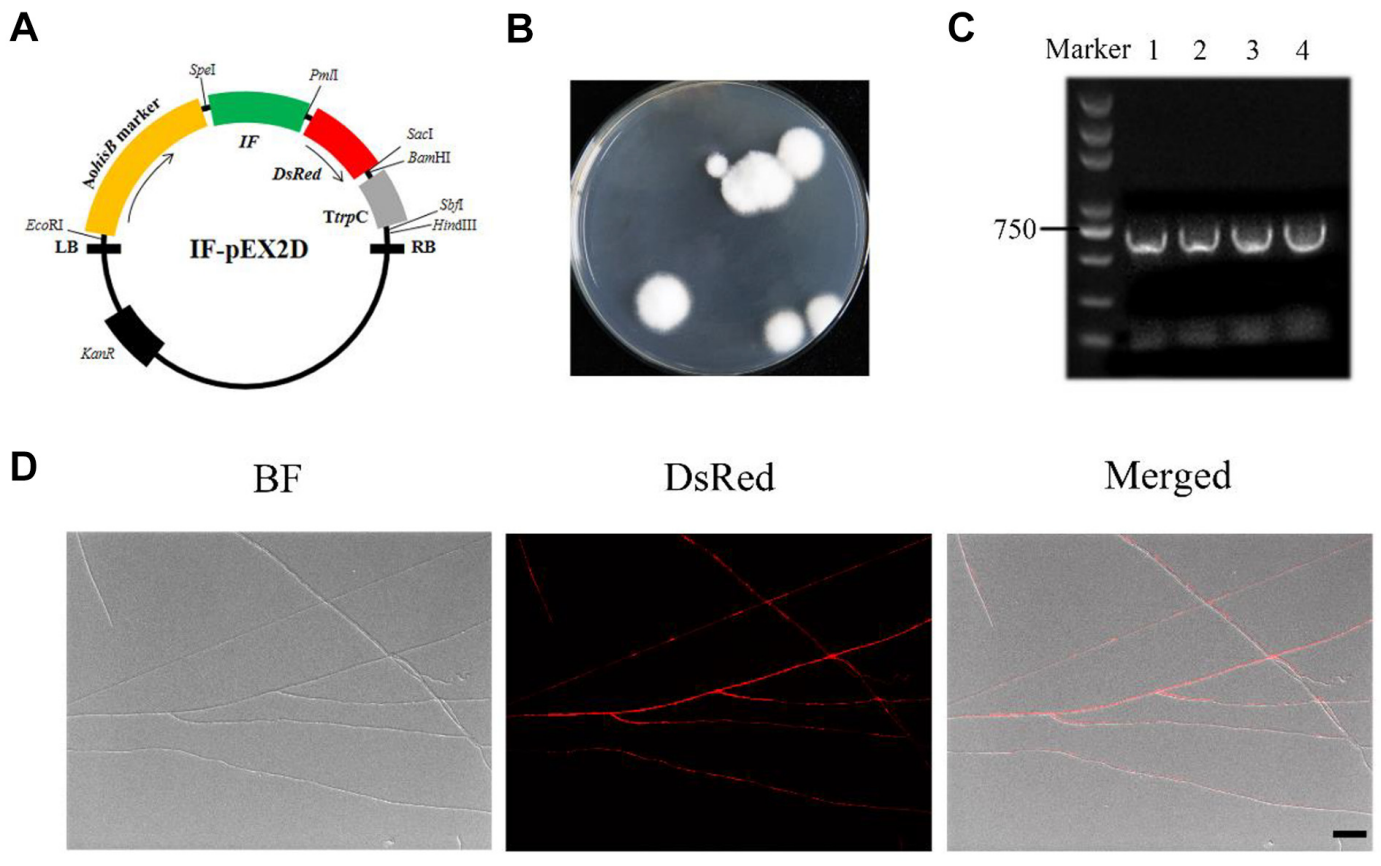
Construction of a transformation system using *hisB* as a selection marker in *C. militaris*. (**A**) The transformation vector IF-pEX2D was obtained by modifying the promoter of the reporter gene in pEX2D. (**B**) Transformants cultured with the IF-pEX2D complementation vector on screening medium for 7 days. (**C**) Primers (*DsRed* F/R) were used to amplify the *DsRed* gene by PCR. Lines 1–4, *DsRed* validation. D: The mycelia of the complementary strains were observed under bright field, fluorescence field, and merged bright field and fluorescence (from left to right). The scale bar represents 5 μm.

The modified vector IF-pEX2D was used to transform the histidine-deficient mutant CmΔ*hisB* by ATMT (Fig. 3A). The vector carrying the *DsRed* fluorescent reporter gene was screened on CD medium without histidine, and the complementation strain with a histidine deficiency was screened (Fig. 3B). The transformation efficiency was 65 ± 25 transformants/10^6^ spores. The transformants were verified by PCR with primers *DsRed* F/R (Fig. 3C) and fluorescence observation (Fig. 3D), revealing that the reporter gene was successfully transformed into *C. militaris* using *hisB* as a selection marker.

### Construction of Double Auxotrophic Mutants in *C. militaris*

**Fig. 4:**
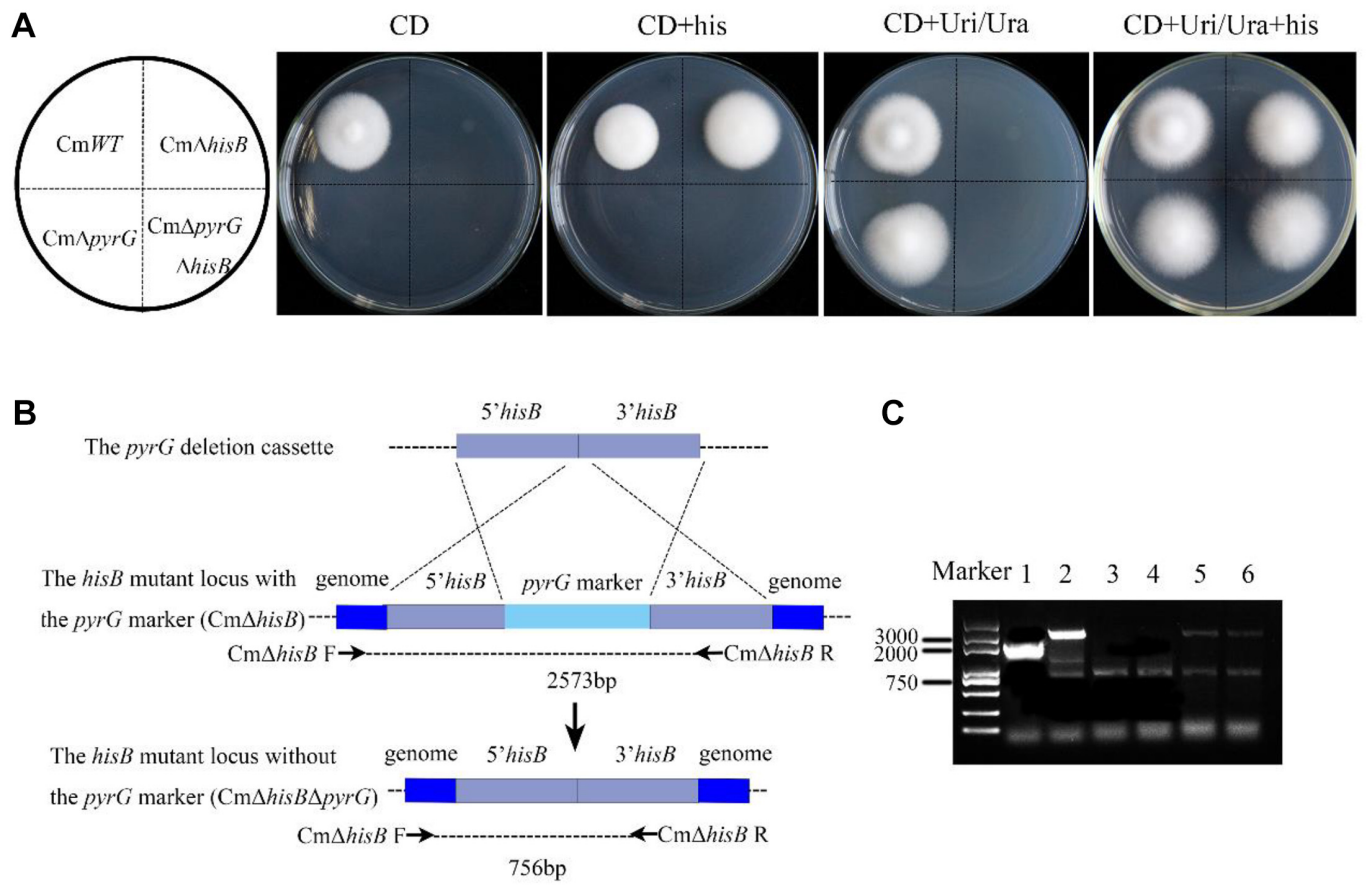
Construction of a dual auxotrophic mutant of *C. militaris*. (**A**) The growth of the dual auxotrophic mutant CmΔ*pyrG*Δ*hisB* was compared with that of wild-type Cm*WT*, Cm*pyrG*, and CmΔ*hisB* on different media. (**B**) Knockout of *pyrG* in the background of the histidine auxotrophic mutant CmΔ*hisB* and the specific primers for verification of the CmΔ*hisB*Δ*pyrG* mutants are indicated. (**C**) Primers (CmΔ*hisB* F/R) were used to verify the size of *hisB* in *C. militaris* mutants: 1 Wild-type strain, 2 Background strain, 3 and 4 Dual auxotrophic mutants, 5 and 6 ectopic strains.

During the construction of histidine auxotrophic *C. militaris*, the *pyrG* expression cassette replaced the ORF of *hisB* in the CmΔ*pyrG* background. Thus, we constructed a CmΔ*pyrG* vector to knock out the transformed *pyrG* expression cassette by homologous recombination (Fig. 4B). The transformants were screened in CD + Uri/Ura + his + 5-FOA medium by homologous recombination. Then, the phenotypes of transformants were validated in CD + his and CD + Uri/Ura + his medium. The phenotypes of the wild-type *C. militaris* (CmWT), uridine/uracil auxotrophic mutant (CmΔ*pyrG*), histidine auxotrophic mutant (CmΔ*hisB*), and dual auxotrophic mutant (CmΔ*pyrG* Δ*hisB*) were analyzed by using CD, CD + Uri/Ura, CD + his, and CD + Uri/Ura + his mediums (Fig. 4A). The dual auxotrophic mutant strain CmΔ*pyrG*Δ*hisB* only grew on medium supplemented with histidine and uridine/uracil (Fig. 4A). The knockout of the *pyrG* gene through homologous recombination was expected to yield a fragment of 756 bp (Fig. 4B). The knockout of the *pyrG* gene was also verified through PCR using the primer CmΔ*hisB* F/R (Fig. 4C). Additionally, a sequencing analysis was implemented to verify the absence of the *pyrG* gene. These findings indicated that the *pyrG* marker was removed from the CmΔ*hisB* genome by homologous recombination, and a dual auxotrophic mutant of *C. militaris* was successfully constructed.

### Establishment of a System for the Simultaneous Transformation of Two Genes

**Fig. 5:**
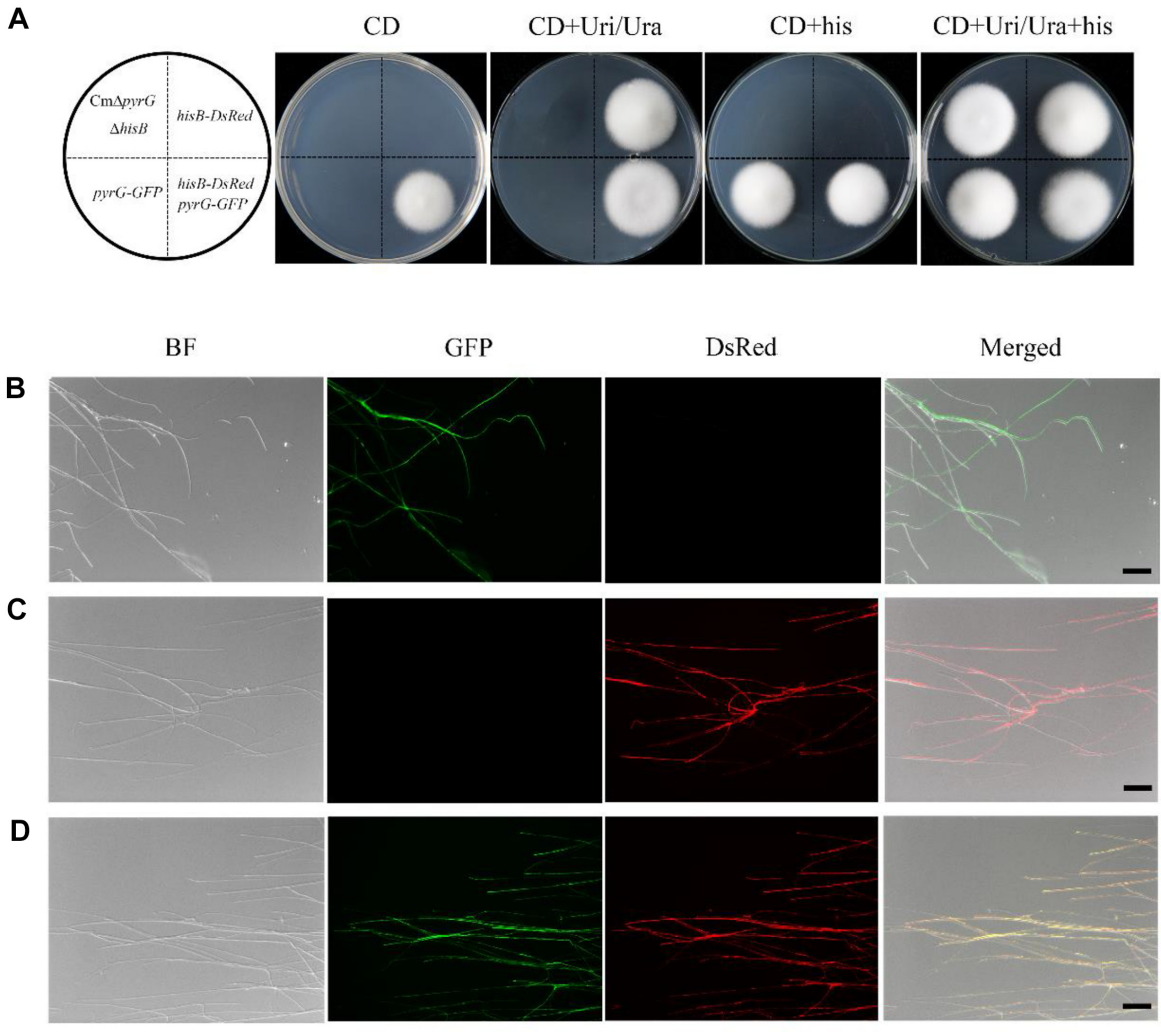
Co-expression of *DsRed* and *GFP* reporter genes in *C. militaris*. (**A**) Phenotype of the dual auxotrophic mutant CmΔ*pyrG* Δ*hisB*, IF-pEX1/CmΔ*pyrG* Δ*hisB*, IF-pEX2D/CmΔ*pyrG* Δ*hisB*, and co-transformed CmΔ*pyrG* Δ*hisB* with IF-pEX1 and IF-pEX2D in different media. (B–D) Fluorescence observation of mycelia from IF-pEX1/CmΔ*pyrG* Δ*hisB*, IFpEX2D/ CmΔ*pyrG* Δ*hisB* and CmΔ*pyrG* Δ*hisB* co-transformed with IF-pEX1 and IF-pEX2D. From left to right, bright field, *GFP* field, *DsRed* field, and bright field superimposed with two fluorescence fields. Scale bar represents 5 μm.

The two binary vectors IF-pEX1 and IF-pEX2D were introduced into the dual auxotrophic mutant CmΔ*pyrG* Δ*hisB* of *C. militaris*. The vector IF-pEX1 carried a *pyrG* expression cassette and *GFP* reporter gene, and the vector IF-pEX2D contained the *hisB* expression cassette and *DsRed* reporter gene. We first transformed IF-pEX1 into CmΔ*pyrG* Δ*hisB*. The transformation efficiency was 75 ± 35/10^6^ spores. Then, the transformants were used as a background for the transformation of IF-pEX2D, with a transformation efficiency of 55 ± 25 transformants/10^6^ spores. The phenotypes of all strains and fluorescence of transformants are shown in Fig. 5. Both IF-pEX1 and IF-pEX2D were transformed into CmΔ*pyrG* Δ*hisB* double auxotrophic mutants, and the reporter genes were successfully expressed.

### Knockout and Complementation of Cm*WC-1* Using the Newly Constructed System

**Fig. 6:**
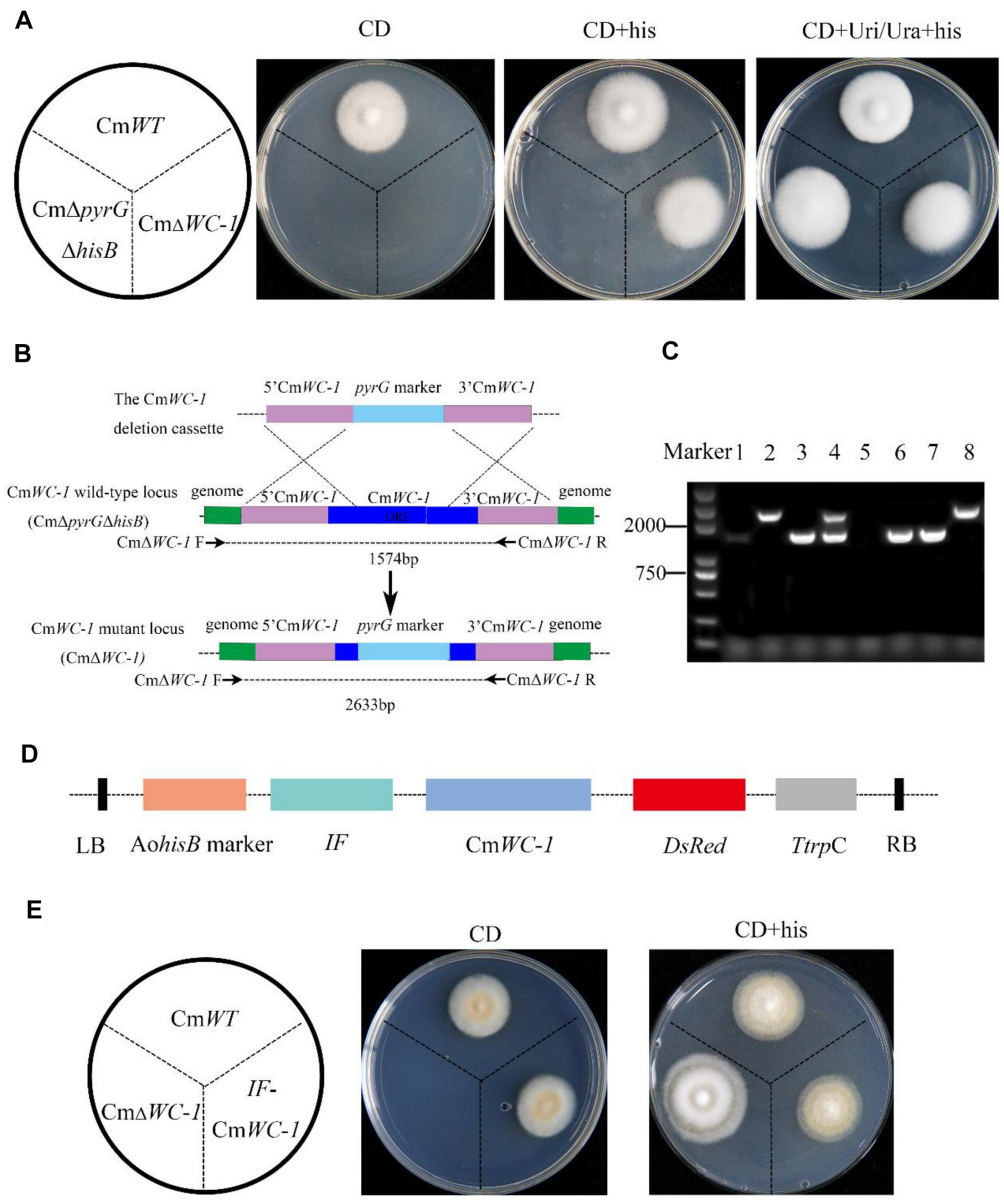
Knockout and complementation of Cm*WC-1*. (**A**) Phenotype of the wild-type CmWT, CmΔ*pyrG*Δ*hisB*, and CmΔWC-1 in different media. (**B**) Scheme for Cm*WC-1* deletion in the dual auxotrophic *C. militaris*. The specific primers for verification of the CmΔWC-1 mutants are indicated. (**C**) PCR verification of the Cm*WC-1* knockout mutant in *C. militaris*: Line 1 Background strain, 2–8 Knockout mutant transformants; lines 2 and 8 represent the knockout mutant, and others represent the ectopic strains. (**D**) Linear diagram of the Cm*WC-1* complementary vector. E: Phenotype of the wild-type CmWT, knockout strain CmΔWC-1, and complementary strain IF-Cm*WC-1* in different media under light.

The knockout of the photoreceptor gene Cm*WC-1* of *C. militaris* results in an albino phenotype when exposed to light [28]. Therefore, to evaluate the efficiency of the dual gene transformation system for assays of gene function, Cm*WC-1* was selected as a target for gene knockout and complementation. A phylogenetic analysis and motif analyses of WC-1 in different species are provided in the supplementary materials (Fig. S1). The Cm*WC-1* gene was knocked out by homologous recombination using *pyrG* as a selection marker in the dual auxotrophic mutant. The transformants were screened on CD + his medium, and the transformation efficiency was (35 ± 15)/ 10^6^ spores. Phenotypes of transformants were confirmed on CD, CD + his, CD + Uri/Ura, and CD + Uri/Ura + his medium (Fig. 6A). Subsequently, the primers CmΔWC-1 F/R were utilized for PCR amplification to detect mutations resulting from homologous recombination. Ectopic recombination resulted in a band size of 1574 bp, while homologous recombination led to a band size of 2633 bp (Fig. 6B). As shown in Fig. 6C, two of the six transformants were confirmed to be CmΔWC-1 knockout strains, and the others were ectopic strains. Sequencing of the homologous arm of Cm*WC-1* further confirmed the successful knockout of Cm*WC-1*. Then, we used IF-pEX2D to transform wild-type Cm*WC-1* for complementation of the CmΔWC-1 mutant strain (Fig. 6D). The phenotypes of the CmΔWC-1 mutant and complementation strains were analyzed. Consistent with previous results, the CmΔWC-1 mutant did not turn yellow under light conditions, while the complementary strain turned yellow, and there was no significant difference in phenotype between the complementary strains and the wild type (Fig. 6E). This analysis supported the value of the dual transformation system as a tool for gene function identification in *C. militaris*.

ATMT has a high transformation efficiency for *C. militaris*. Each transformation plate can contain 2–6 transformants. Although the transformation cycle is longer than that of most filamentous fungi, the transformation efficiency can reach 100%. Accordingly, this method is commonly used in *C. militaris*. For example, a *C. militaris* g38 mutant with a short culture time, reduced airborne mycelium, fast stratigraphy differentiation, and high fruiting body yield was isolated by the ATMT method [29]. Additionally, the ATMT method was used to construct a *C. militaris* strain expressing *cas9* [30]. To study the function of Cm*vvd* in *C. militaris*, the gene was disrupted by homologous recombination using ATMT [28]. ATMT was used to explore the roles of the Cm*Snf1* gene [31] as the ben gene in *C. militaris* [32]. The ATMT method provides technical support for investigating gene functions in *C. militaris* and elucidating biosynthetic pathways of its active components.

The *pyrG* auxotrophic *C. militaris* has been generated by our laboratory [27]. However, for studies of gene function, gene deletion and complementation require two different selection markers. In addition, a genetic system using *hisB* as a selection marker is currently not available for *C. militaris*. In this study, CmΔ*pyrG* was used as a background strain to knock out *hisB* in *C. militaris* by ATMT. Then, *hisB* of *C. militaris* was replaced with a binary vector containing *pyrG* of *A. oryzae*, and a stable auxotrophic mutant CmΔ*hisB* was obtained. This mutant was transformed with a vector containing *pyrG* of *A. oryzae*, and *C. militaris* with the *DsRed* marker was obtained successfully, indicating that *hisB* in *A. oryzae* could complement the *hisB* auxotrophic mutant of *C. militaris*, and the function of *hisB* was relatively conserved. Based on the CmΔ*hisB* mutant, *pyrG* was knocked out by homologous recombination in *C. militaris* with a binary vector containing upstream and downstream regions of *hisB*, and a dual auxotrophic strain CmΔ*pyrG*Δ*hisB* was obtained successfully. Furthermore, *hisB* and *pyrG* were successfully inserted in the CmΔ*pyrG*Δ*hisB* mutant by ATMT, and *DsRed* and *GFP* reporter genes were expressed simultaneously in *C. militaris*. The genetic transformation system with *hisB* and *pyrG* as screening markers was successfully established.

In addition, we used the Cm*WC-1* gene to detect the knockout and complementation efficiency of the ATMT system. *C. militaris* changes from white to yellow or orange in light [33]. Inactivation of the Cm*WC-1* gene leads to the thickening of aerial hyphae and significant reductions in the production of carotenoid and cordycepin, leading to a significant reduction of pigmentation [34]. The albino phenotype was clearly observed when Cm*WC-1* was disrupted by homologous recombination using ATMT, and *C. militaris* strains lacking Cm*WC-1* show faster growth than that of wild-type strains under light [30, 35]. In this study, the Cm*WC-1* gene was successfully knocked out by the ATMT system using the dual auxotrophic mutant of *C. militaris* as a background strain, with an efficiency of 33.3%, and the phenotype of the knockout strain was consistent with previous results. These findings indicate that the two screening markers are useful for studies of gene function, with safety and a high transformation efficiency. In general, the newly constructed ATMT system can be used to test the functions of target genes and provides a convenient approach for studies of recombinant gene expression in *C. militaris*.

## Supplemental Materials

Supplementary data for this paper are available on-line only at http://jmb.or.kr.



## References

[1] Das SK, Masuda M, Sakurai A, Sakakibara M (2010). Medicinal uses of the mushroom *Cordyceps militaris*: current state and prospects. Fitoterapia..

[2] Gu YX, Wang ZS, Li SX, Yuan QS (2007). Effect of multiple factors on accumulation of nucleosides and bases in *Cordyceps militaris*. Food Chem..

[3] Ng TB, Wang HX (2005). Pharmacological actions of Cordyceps, a prized folk medicine. J. Pharm. Pharmacol..

[4] Yang L, Li G, Chai Z, Gong Q, Guo J (2020). Synthesis of cordycepin: current scenario and future perspectives. Fungal Genet. Biol..

[5] Wang L, Yan H, Zeng B, Hu Z (2022). Research progress on cordycepin synthesis and methods for enhancement of cordycepin production in *Cordyceps militaris*. Bioengineering (Basel).

[6] Phull AR, Ahmed M, Park HJ (2022). *Cordyceps militaris* as a bio functional food source: pharmacological potential, antiinflammatory actions and related molecular mechanisms. Microorganisms.

[7] Seong da B, Hong S, Muthusami S, Kim WD, Yu JR, Park WY (2016). Cordycepin increases radiosensitivity in cervical cancer cells by overriding or prolonging radiation-induced G2/M arrest. Eur. J. Pharmacol..

[8] Nxumalo W, Elateeq AA, Sun Y (2020). Can Cordyceps cicadae be used as an alternative to *Cordyceps militaris* and *Cordyceps sinensis*? - a review. J. Ethnopharmacol..

[9] Kim HO, Yun JW (2005). A comparative study on the production of exopolysaccharides between two entomopathogenic fungi *Cordyceps militaris* and *Cordyceps sinensis* in submerged mycelial cultures. J. Appl. Microbiol..

[10] Khan MA, Tania M (2020). Cordycepin in anticancer research: molecular mechanism of therapeutic effects. Curr. Med. Chem..

[11] Zheng P, Xia Y, Xiao G, Xiong C, Hu X, Zhang S (2011). Genome sequence of the insect pathogenic fungus *Cordyceps militaris*, a valued traditional Chinese medicine. Genome Biol..

[12] Zheng Z, Huang C, Cao L, Xie C, Han R (2011). *Agrobacterium tumefaciens*-mediated transformation as a tool for insertional mutagenesis in medicinal fungus *Cordyceps militaris*. Fungal Biol..

[13] de Bekker C, Wiebenga A, Aguilar G, Wosten HA (2009). An enzyme cocktail for efficient protoplast formation in *Aspergillus niger*. J. Microbiol. Methods.

[14] Ruiz-Diez B (2002). Strategies for the transformation of filamentous fungi. J. Appl. Microbiol..

[15] Schmoll M, Zeilinger S (2021). Resistance marker- and gene gun-mediated transformation of *Trichoderma reesei*. Methods Mol. Biol..

[16] Li D, Tang Y, Lin J, Cai W (2017). Methods for genetic transformation of filamentous fungi. Microb. Cell Fact..

[17] Jiang D, Zhu W, Wang Y, Sun C, Zhang KQ, Yang J (2013). Molecular tools for functional genomics in filamentous fungi: recent advances and new strategies. Biotechnol. Adv..

[18] Idnurm A, Bailey AM, Cairns TC, Elliott CE, Foster GD, Ianiri G (2017). A silver bullet in a golden age of functional genomics: the impact of *Agrobacterium*-mediated transformation of fungi. Fungal Biol. Biotechnol..

[19] Lou H, Zhao Y, Zhao R, Ye Z, Lin J, Guo L (2021). Screening and functional verification of selectable marker genes for *Cordyceps militaris*. J. Food Qual..

[20] Weidner G, d'Enfert C, Koch A, Mol PC, Brakhage AA (1998). Development of a homologous transformation system for the human pathogenic fungus *Aspergillus fumigatus* based on the *pyrG* gene encoding orotidine 5'-monophosphate decarboxylase. Curr. Genet..

[21] Skory CD, Horng JS, Pestka JJ, Linz JE (1990). Transformation of *Aspergillus parasiticus* with a homologous gene (*pyrG*) involved in pyrimidine biosynthesis. Appl. Environ. Microbiol..

[22] Nguyen KT, Ho QN, Pham TH, Phan TN, Tran VT (2016). The construction and use of versatile binary vectors carrying *pyrG* auxotrophic marker and fluorescent reporter genes for *Agrobacterium*-mediated transformation of *Aspergillus oryzae*. World J. Microbiol. Biotechnol..

[23] Nguyen KT, Ho QN, Do L, Mai LTD, Pham DN, Tran HTT (2017). A new and efficient approach for construction of uridine/uracil auxotrophic mutants in the filamentous fungus *Aspergillus oryzae* using *Agrobacterium tumefaciens*-mediated transformation. World J. Microbiol. Biotechnol..

[24] Du Y, Xie G, Yang C, Fang B, Chen H (2014). Construction of brewing-wine *Aspergillus oryzae*
*pyrG*- mutant by *pyrG* gene deletion and its application in homology transformation. Acta Biochim. Biophys. Sin (Shanghai)..

[25] Fiedler MR, Gensheimer T, Kubisch C, Meyer V (2017). *HisB* as novel selection marker for gene targeting approaches in *Aspergillus niger*. BMC Microbiol..

[26] Thai HD, Nguyen BT, Nguyen VM, Nguyen QH, Tran VT (2021). Development of a new *Agrobacterium*-mediated transformation system based on a dual auxotrophic approach in the filamentous fungus *Aspergillus oryzae*. World J. Microbiol. Biotechnol..

[27] Wang L, Huang H, Liu XP, Wang XM, Zeng B, Hu ZH (2022). Construction of *Agrobacterium*-mediated auxotrophic strain and genetic transformation system of *Cordyceps militaris*. Microbiol. China..

[28] Zhang J, Wang F, Yang Y, Wang Y, Dong C (2020). *CmVVD* is involved in fruiting body development and carotenoid production and the transcriptional linkage among three blue-light receptors in edible fungus *Cordyceps militaris*. Environ. Microbiol..

[29] Jiang K, Han R (2015). *Rhf1* gene is involved in the fruiting body production of *Cordyceps militaris* fungus. J. Ind. Microbiol Biotechnol..

[30] Chen BX, Wei T, Ye ZW, Yun F, Kang LZ, Tang HB (2018). Efficient CRISPR-Cas9 gene disruption system in edible-medicinal mushroom *Cordyceps militaris*. Front. Microbiol..

[31] Wang Y, Wang R, Wang Y, Li Y, Yang RH, Gong M (2020). Diverse function and regulation of *CmSnf1* in entomopathogenic fungus *Cordyceps militaris*. Fungal Genet. Biol..

[32] Lou HW, Zhao Y, Ren CS, Zhao RY, Ye ZW, Lin JF (2021). Cloning of the *ben* gene and its functional identification in *Cordyceps militaris*. Scientia Horticulturae..

[33] Yang T, Dong C (2014). Photo morphogenesis and photo response of the blue-light receptor gene *Cmwc-1* in different strains of *Cordyceps militaris*. FEMS Microbiol Lett..

[34] Yang T, Guo M, Yang H, Guo S, Dong C (2016). The blue-light receptor *CmWC-1* mediates fruit body development and secondary metabolism in *Cordyceps militaris*. Appl. Microbiol. Biotechnol..

[35] Meng G, Wang X, Liu M, Wang F, Liu Q, Dong C (2022). Efficient CRISPR/Cas9 system based on autonomously replicating plasmid with an AMA1 sequence and precisely targeted gene deletion in the edible fungus, *Cordyceps militaris*. Microb. Biotechnol..

