# Supplementary material for: Construction of a New Agrobacterium tumefaciens-Mediated Transformation System based on a Dual Auxotrophic Approach in Cordyceps militaris
Source: J Microbiol Biotechnol. 2024 Mar 18;34(5):1178–87. doi: 10.4014/jmb.2312.12003 (PMC11180907; doi:10.4014/jmb.2312.12003)
Supplement: Supplementary file 1 [file jmb-34-5-1178-supple.pdf]

J. Microbiol. Biotechnol. 2024. 34(5): 1178–1187
https://doi.org/10.4014/jmb.2312.12003

Supplementary Figure and Tables

Construction of a new *Agrobacterium tumefaciens*-mediated transformation system based on a dual auxotrophic approach in *Cordyceps militaris*

Huan huan Yan[1,2], Yi tong Shang[1,2], Li hong Wang[1,2], Xue qin Tian[1,2], Van-Tuan Tran[4], Li hua Yao[1], Bin Zeng[3*], Zhi hong Hu[1,2*]

[1]College of Life Sciences, Jiangxi Science and Technology Normal University, Nanchang 330013, P. R. China

[2]Jiangxi Key Laboratory of Bioprocess Engineering, Jiangxi Science and Technology Normal University, Nanchang 330013, P. R. China

[3]Shenzhen Technology University, Shenzhen 518118, P. R. China

[4]VNU University of Science, 334 Nguyen Trai, Thanh Xuan, Hanoi, Vietnam

***Correspondence author: Zhihong Hu**, huzhihong426@163.com;

C. militaris possesses a conserved *WC-1* gene in the genome

Neighbor-joining method was used to create the unrooted tree using MEGA-X, and MEME program was used to identify the conserved motifs of all proteins. The amino acid sequence used is as follows, *Cordyceps militaris* (AGO64764.1), *Cordyceps cicadae* (WAI89161.1), *Beauveria bassiana* (KAH8718004.1), *Aphanocladium album* (KAJ6788919.1), *Purpureocillium lilacinum* (GJN82470.1) and *Fusarium austroafricanum* (KAF4456728.1). The results confirm that the evolutionary process of *WC-1* is conserved in different species.

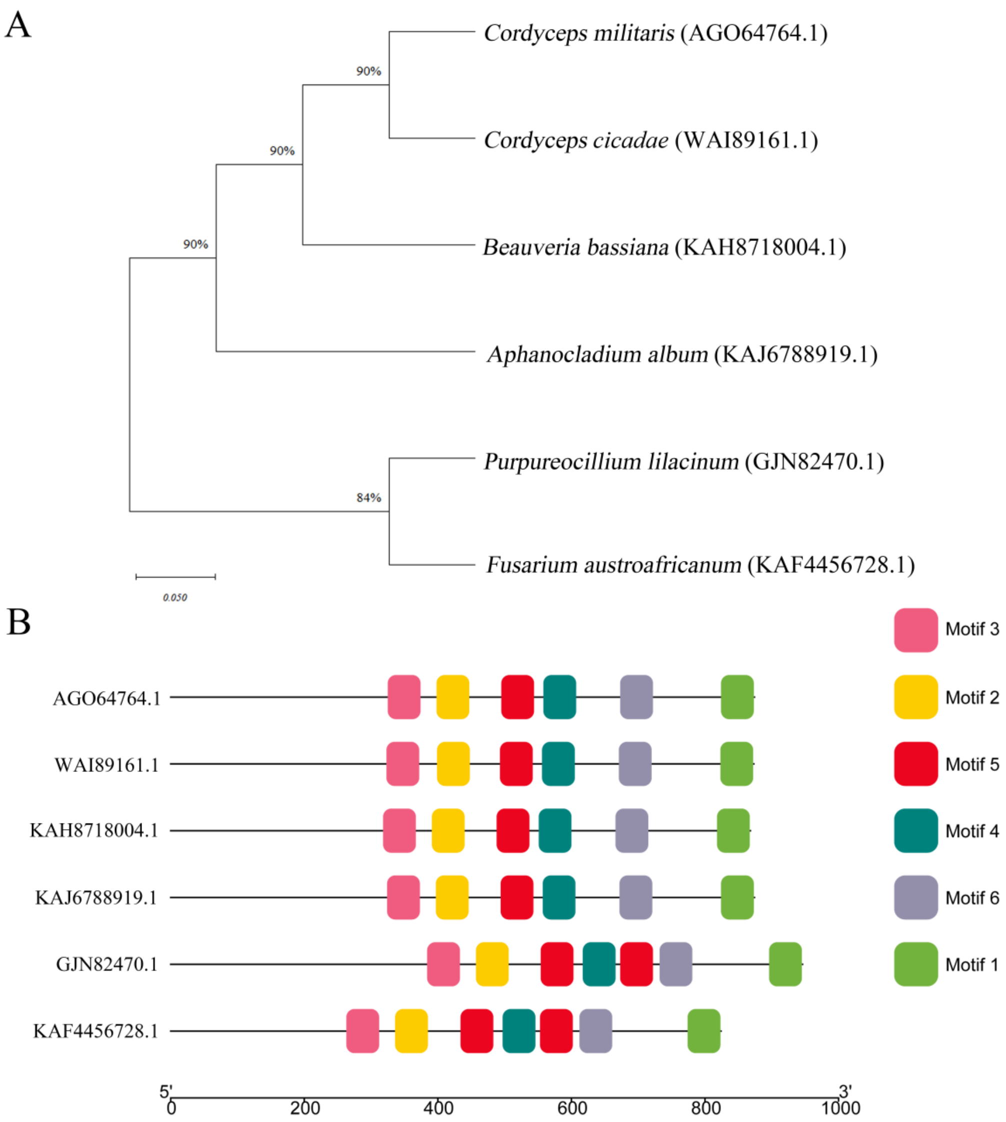

Fig. S1. Phylogenetic analysis and functional motifs of *WC-1* in different species. (A) Unrooted phylogenetic tree of *WC-1* and homologous proteins in *Cordyceps militaris*, *Cordyceps cicadae*, *Beauveria bassiana*, *Aphanocladium album*, *Purpureocillium lilacinum* and *Fusarium austroafricanum*. The IDs of the sequences were included after the specie names in the figure; **(B)** All conserved motifs of the *WC-1* were identified by the MEME program. Protein sequences are indicated by thin black line, and the conserved motifs are represented by different colored boxes.

Table S1. Strains and plasmids for this work.

Strains and plasmids	Description	Sources
E. coli DH5α	Cloning host	This lab
A. tumefaciens AGL1	As a tool to transfer genes into C. militaris cells	This lab
C. militaris	wild-type strain, isolated from production extraction	This lab
pEX1	carries *pyrG* cassette and *GFP* gene	provide by Professor Van-Tuan Tran
pEX2D	carries *his* cassette and *DsRed* gene	provide by Professor Van-Tuan Tran
CmΔ*pyrG*	Stored by our laboratory	This lab
CmΔ*his*	used to delete *his* gene	This study
IF-pEX2D	used to express the red fluorescent protein	This study
IF-pEX1	used to express the green fluorescent protein	This lab
CmΔ*WC-1*	used to delete the Cm*WC-1* gene	This study
IF-Cm*WC-1*	used to complement the Cm*WC-1* gene	This study

Table S2. Primers for this study.

Primers	Sequences (5′→3′)	Sizes (bp)	Description
hisB upstream F1(same as F4)	TATGACATGATTACGAATTCGTGTGGTAGCGACGATTTAG	1294	Deletion of *hisB* in C. militaris using the *pyrG* selectable marker
hisB upstream R1(same as R4)	ACTTACCTTCGCATGAATTCGCGTATGTGCGTTAGATACTC		
hisB downstream F2	TCAGAGCCTAGCCAACTAGTGGGAGATTTTGAGTTCGATG	1244	
hisB downstream R2 (same as R3)	CGACGGCCAGTGCCAAGCTTGTAGGATTCAATGACCTGC		
hisB upstream F3	TATGACATGATTACGAATTCGGGAGATTTTGAGTTCGATG	1294	removal of the *pyrG* marker from genomes of the histidine auxotrophic mutants
CmΔ*hisB* F	ACTTTTTGGCTTGTGGTGC	742	Verify deletion of *hisB* gene
CmΔ*hisB* R	GCGGGTATCAAGCTCTAGAC		
IF-pEX2D F	CTTGTCCCGGTACGACTAGTTTCGGCAAGAATAGGAACAG	1602	The promoter of the vector was changed to express red fluorescence
IF-pEX2D R	ATGATATCCTTAAGCACGTGTTTGAAGGTGTTTGTTATTG		
IF-pEX1 F	TCAGAGCCTAGCCAACTAGTTTCGGCAAGAATAGGAACAG	1602	The promoter of the vector was changed to express green fluorescence
IF-pEX1 R	ATGGTACCTACGTACTCGAGTTTGAAGGTGTTTGTTATTG		
GFP F	GTGAGCAAGGGCGAGGAGCTGTTCACCG	714	Verify *GFP*
GFP R	CTTGTACAGCTCGTCCATGCCGTGAGTG		
DsRed F	ATGGCCTCCTCCGAGGACG	675	Verify *DsRed*
DsRed R	CAGGAACAGGTGGTGGCGG		
Cm*WC-1* upstream F	TATGACATGATTACGAATTCTTTATTGGGACCTCTAACTA	1514	Deletion of Cm*WC-1* in *C. militaris*
Cm*WC-1* upstream R	ACTTACCTTCGCATGAATTCTGTCATGGCTACTGTACCCA		
Cm*WC-1* downstream F	TCAGAGCCTAGCCAACTAGTAAATGCATCATTCTTGTCGG	1313	
Cm*WC-1* downstream R	CGACGGCCAGTGCCAAGCTTCATCACATCAGCGCAAAACT		
CmΔ*WC-1* F	ATGCAGCTTTCTATGTCCCC	1574	Verify deletion of Cm*WC-1* gene

CmΔ*WC-1* R	TTGAGGACTTCGTGTTTGCA		
IF-Cm*WC-1* F	CAAACACCTTCAAACACGTGA TGGAAGGCTACTATCCTCC	2890	used to complement the Cm*WC-1* gene
IF-Cm*WC-1* R	ATGATATCCTTAAGCACGTGAG TCGAGCTAGTCTCTCGCT		
